#  Chromatin Diminution Process Regulates rRNA Gene Copy Number in Freshwater Copepods 

**Published:** 2010

**Authors:** M. V. Zagoskin, T. L. Marshak, D. V. Mukha, A. K. Grishanin

**Affiliations:** Vavilov Institute of General Genetics, Russian Academy of Sciences; Koltsov Institute of Developmental Biology, Russian Academy of Sciences

**Keywords:** Chromatin diminution, ribosomal RNA genes, Copepoda, real-time PCR, gene copy number

## Abstract

The results of quantitative PCR (qPCR) presented in the paper clearly demonstrate that the sixteen-fold genome reduction in*Cyclops kolensis*during chromatin diminution (from 15.3 pg to 0.98 pg) results in a dramatic decrease in ribosomal RNA gene copy numbers in the genome of a somatic cell line by more than two orders of magnitude. The results presented allow for the consideration of the chromatin diminution as a mechanism of rDNA copy number regulation.

##  INTRODUCTION 

Chromatin diminution (CD) is the programmed process of elimination of a considerable fraction of chromatin from the genome of somatic precursor cells and occurs during early developmental stages of some multicellular eukaryotes, or from the somatic nucleus (the macronucleus) during its formation in protozoa. Knowledge of the phenomenon of chromatin diminution has existed for over a hundred years. Yet, it has been established to exist only in less than 100 belonging to only a few taxa: including protozoa, nematodes, and copepods [1, 2]. CD has been described in approximately 20 cyclop species [1].

The rRNA genes in the genome of most eukaryote species are represented by a large copy number and organized as cistrons. Copies of the ribosomal cistron are repeated in tandem form in one or several clusters, which may be located on one or several chromosomes [3]. A large amount of data, mainly concerning the structural-functional organization of rRNA genes, has been accumulated over the past decades [4–6]. Recent studies have been directed primarily towards the investigation of the mechanisms controlling the regulation of the transcriptional activity of rRNA genes. It should be noted that rRNA molecules represent more than half of all RNA synthesized in a cell [6], and rRNA genes are responsible for approximately 35–60% of the total transcriptional activity in a cell [7].

It is known that the copy number of rRNA genes in the eucaryotic genome varies over an appreciably wide range: from 39 to 19,300 in animals and from 150 to 26,048 in plants [8]. A number of cases have been described with a variation of the rDNA copy number, including an increase in the amount of rDNA due to the amplification of extrachromosomal rRNA gene copies in *Xenopus laevis* oocytes [9–11] or in protozoa [12]. The rRNA gene number may also decrease, as occurs in *Drosophila melanogaster * with the so-called *bobbed (bb)* mutation; however, the number of rDNA repetitions completely recovers by the third–fourth generations [13–18]. Although this variation in the rDNA copy number in the genome is likely an exception, it suggests the existence and maintenance of mechanisms for regulation of the rRNA gene copy number at a certain level.

The programmed gene elimination during onthogenesis results from CD as well, although this has been detected so far only in two nematode species: *Ascaris lumbricoides * and * A. suum* . In *A. lumbricoides* , the gene encoding the ALEP-1 ribosomal protein is cleaved [19], whereas the three unique genes ( *rpS19G* , *fert-1* , *aleg-3) * and a retransposon ( *Tas)* [20–22]) that are eliminated by CD have been detected thus far only in *A. suum* . The remaining portion of the eliminated sequences being eliminated are noncoding. It has been repeatedly noted that CD could be an informative model for studying the excessive DNA problem and genome reorganization during ontogenesis. However, the significance of CD has been underappreciated because of the lack of data [23, 24].

Despite the fact that 94% of DNA is eliminated in *Cyclops kolensis * during CD [25], until recently, only either noncoding nucleotide sequences enriched in repeat regions or satellite DNA have been detected in the fraction eliminated from the genome [26–28]. Earlier, it was assumed that there is a possibility of elimination of a fraction of ribosmal cistrons from *C. kolensis * chromosomes as a result of CD [29]. Prokopovich *et al* . have noted the positive correlation between the genome’s size and the rDNA copy number [8].

This study was aimed at ascertaining the ratio of rRNA gene copy number in prediminuted to post-diminuted *C. kolensis * genomes and estimating the rRNA gene copy number in the Russian population of *C. insignis* , the species which lacks CD [30]. For this purpose, we used quantitative PCR (qPCR), along with other techniques for determining gene copy number. For the present purposes, qPCR is the most efficient and informative method [31–33].

##  EXPERIMENTAL 

Cyclops *C. kolensis * Lill *. * and *C. insignis * Claus were collected from a pond in the Vorob’evy Gory, Moscow, Russia (55°42’35.40”N; 37°34’6.61”W) in April 2009–2010.

**Cytophotometry**

The procedure [25, 34] for the preparation of specimens of embryonic and somatic cells of adult *C. kolensis, * in order to carry out Feulgen staining, was subjected to modifications in which destruction of the shells of embryo sac and egg was performed. Sperm cells and erythrocytes of loach were used as an internal control for determining the absolute amount of DNA. The DNA content in loach ( *Misgurnus fossilis* ) cells is 1C = 2.4 pg [35]. Loach sperm cells contain 1C = 2.4 ± 0.2 (SD) pg of DNA; for erythrocytes, 2C = 5.1 ± 0.4 (SD) pg. The DNA content was measured in 100 erythrocytes and 127 sperm cells of loach. The procedure of sperm smear preparation included the trituration of the male gonads of the loach in the standard physiological solution – 0,9% NaCl; the resulting cell suspension was used to prepare the smears. After drying, the specimens were immobilized for 10 minutes in 96% ethanol, at room temperature. Cyclops were fixed in an ethanol–acetic acid mixture (3 : 1), squashed in 45% acetic acid to completely separate the shells of the embryo sac and embryo itself. Next, they were subjected to the Feulgen staining procedure in order to measure the amount of DNA. Feulgen staining of the cell nuclei was carried out under the following conditions: hydrolysis in 5 N HCl for 11 min at 37°С and staining with Schiff’s reagent (1 hr at room temperature). The measurements were carried out on a Vickers M86 microdensitometer (England) (wavelength of 540 nm). All specimens used for the measurements were processed in the same staining batch. Fifty-nine cells of the second polar body were analyzed at the metaphase state in the pre-diminution genome and 140 somatic line cells of an adult cyclops after CD. The results were processed using Microsoft Excel 2007 software (the descriptive statistics).

**Data collection and DNA isolation**

The *C. kolensis* embryo sacs with embryos at stages of four to eight cells were taken as the pre-diminution material. The selection of the embryos was carried out according to the aforementioned procedure [25], which allowed *in vivo* determination of the cell division stage of cyclops embryos using a light microscope. The *C. kolensis* antennae, comprising somatic cells only, served as the post-diminution material. Two antennae were severed with a scalpel from each individual and placed on a glass slide located on a liquid-nitrogen cooled table. The embryo sacs were immobilized in liquid nitrogen. The *C. insignis * individuals were selected because no CD was observed in their ontogenesis.

Since there was a very small amount of DNA material available, genomic DNA was isolated using the Diatom ^TM^ DNA Prep 100 reagent kit (Izogen, Moscow). This kit makes it possible to minimize DNA loss during isolating. Its operating principle is based on the lysis of a specimen in guanidine thiocyanate (a strong chaotropic agent) followed by DNA sorption on silica gel. The material was prehomogenized in a buffer solution (0.2 M Tris, 50 mM EDTA, 0.5% SDS, 200 µg/ml of proteinase K) and lyzed for 1 h at 50°С. The lysate was treated with RNase (0.1 mg/ml) for 5 min; DNA was then isolated according to the procedure recommended by the manufacturer.

**Real-time PCR using EVA Green dye**

Concentrations of the total *C. kolensis* DNA before and after CD and the total *C. insignis * DNA were determined on a Nanodrop 1000 spectrophotometer. The coefficient of variation of DNA concentration was calculated on the basis of two replicates and was equal to 1.33% (before CD) and 5.91% (after CD) in *C. kolensis* and 9.18% in * C. insignis.*

For carrying out real-time PCR, specific primers were constructed (28real_for – 5’-Ggtagccaaatgcctcgtc-3’ and 28real_rev– 5’-CGCCAAAGATGCTCCGCCAC-3’), which allowed the amplification of the 183 bp fragment of the 28S rRNA gene. The fragment length of the 28S rRNA gene was equal in C *. kolensis* and  *C. insignis* .

 Real-time PCR was carried out on an iCycler iQ4 amplificator (Bio-Rad, United States). The data were calculated using iQ5 Optical System Software. The threshold value of accumulation of the amplification products was determined by visual analysis of the PCR product accumulation curves. This value lay within the region of exponential growth of the curves and was equal to 100 in all calculations. 

The real-time PCR was carried out using the “Reaction mixture 2.5x for carrying out real-time PCR in the presence of EVA green dye and ROX reference dye” kit (Sintol, Russia). According to the manufacturer’s recommendations, the reaction was carried out in a volume of 25 µl: 11 µl of PCR standard water, 10 µl of the finished reaction mixture (including deoxynucleoside triphosphates, PCR buffer, MgCl _2_ , and Taq DNA polymerase with antibodies inhibiting enzymatic activity), 1 µl of each primer (the final concentration was 0.4 pmol/µl), and 2 µl of the DNA matrix. The conditions during real-time PCR were as follows: primary denaturation for 4.5 min at 95°C, followed by 50 cycles: 95°С – 15 s, 64°С – 15 s, 72°С – 20 s. The fluorescent signal was recorded at the annealing stage at 64°С. After the amplification, a fusion curve with the 0.5°С temperature gradient (from 55 to 94.5°С) was built, which attested to the presence of only one specific amplification product in each specimen.

**Plasmid DNA preparation**

The PCR product of the 28S gene in *C.*   *kolensis* rRNA, with a length of 2,199 bp, was cloned into the pGEM-T Easy vector (Promega, United States), yielding pGEM-20b1 plasmid. Plasmid DNA (pDNA) was purified on columns using the Wizard Plus SV Minipreps DNA Purification System kit (Promega, United States), according to the manufacturer’s recommendations and including treatment with RNase A. In order to approach the conditions of amplification of the linear DNA, the circular pDNA was cleaved by *Pst* I restriction enzyme, which recognizes the plasmid polylinker and is absent in the inserted fragment. pGEM-20b1 plasmid (2.6 µg) was treated with 2 µl of *Pst* I restrictase (Fermentas) in a 50 µl volume: 23 µl of purified water, 5 µl of 10×Buffer, and 20 µl of pDNA. After incubation for 3 h at 37°С, the plasmid was purified using the phenol-chloroform method and dissolved in the same buffer as the specimens of the genome DNA.

**Construction of the calibration curve to determine the rDNA copy number**

A calibration curve was used in the method of absolute determination of the rRNA gene copy number. A series of five-fold dilutions of pGEM-20b1/ *Pst* I (from 1 ng to 0.32 pg) was used to construct the curve. Each dilution of pDNA was carried out using two replicates. The initial plasmid concentration was measured on a Nanodrop 1000 spectrophotometer with four replicates; the coefficient of variation was equal to 2.36%. The calibration curve had the following characteristics: the coefficient of correlation ( *R* ^2^ )  – 0.996; slope of the curve = -3.760; efficiency ( *E* ) – 84.5% (Figure).

The size of the plasmid with an inserted fragment is 5216 bp. Based on the nucleotide’s composition, the molar mass of the plasmid in double-stranded form was determined using OligoII Mass Calculator v.1.0 software (М = 3.23 × 10 ^6^  g/mol). The number of plasmid DNA molecules was calculated using formula (1):


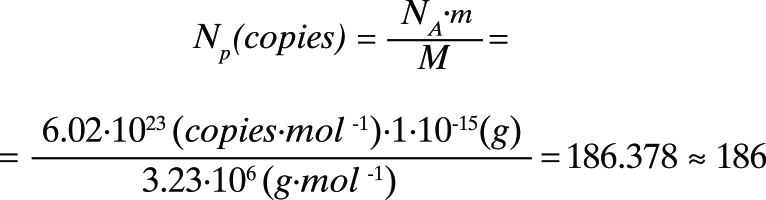
, (1)

where *N _p_* is the number of plasmid DNA molecules per 1 fg of pDNA; *N _A_* is the Avogadro constant; *m* is the DNA amount for which the copy number is calculated; and *М* is the molar mass of a plasmid. It is shown that approximately 186 pDNA molecules correspond to 1 fg of pDNA.

##  RESULTS 

**Cytophotometry**

It was previously shown that the haploid genome of embryomatic and somatic cells of adult *C.*   *insignis * contains 2.1–2.15 pg of DNA [30].

**Fig. 1:**
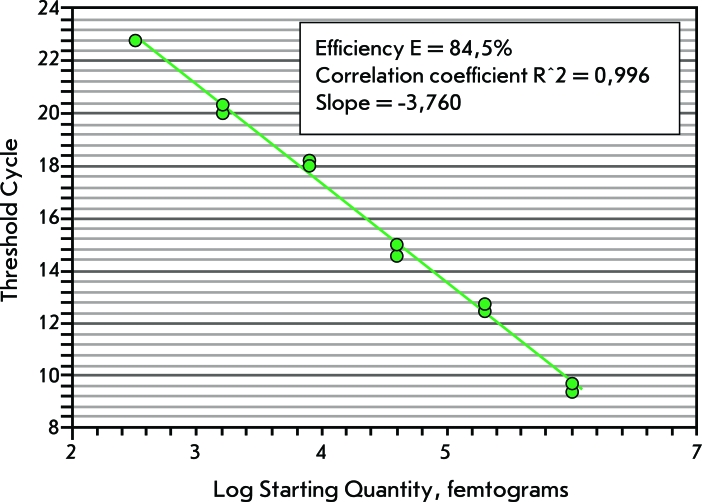
The calibration curve, which was used in the method of absolute determination of the rRNA gene copy number.

During the initial stages of the study of CD, we experienced certain methodological difficulties in the preparation of *C.*   *kolensis * specimens for cytophotometry [25, 36]. Therefore, in order to calculate the rRNA gene copy number in *C.*   *kolensis * cells before and after CD, we repeatedly measured the DNA amount in *C.*   *kolensis * cells by quantitative cytophotometry. The measurement results attest to the change in the absolute DNA amount in *C.*   *kolensis * cells before and after CD. The pre-diminuted genome contained 1С = 15.3 ± 3.1 (SD) pg of nuclear DNA, while the somatic line cells after CD at the anaphase stage contained 0.98 ± 0.13 (SD) pg in equivalence to the haploid genome. The relative amount of DNA being eliminated remained constant and was equal to 94%, which is consisted with published reports [25, 34]. The results of cytophotometric measurements make it possible to estimate the number of cells corresponding to different stages of development in *C.*   *kolensis* and *C.*   *insignis* .

**Determination of the relative rRNA gene copy number using the**

**2 ^–∆C’T ^ method [37]**

No nucleotide sequences of any genes in the species subjected to study were known at the time this research was carried out; thus it was not possible to perform the experiment with this type of internal control. Accordingly, the external standardization was carried out relative to the amount of DNA at the beginning of the reaction; i.e., the equal amounts of *C.*   *kolensis * DNA before and after CD and *С.*   *insignis* DNA were compared. The reactions were performed with 500, 400, 300, and 200 pg dilutions for each DNA specimen. Each reaction with a particular starting amount of DNA was represented in three replicates. When determining the relative amounts using one gene, the 2 ^–∆C’T ^ method is used for processing the PCR results. The specimens with post-diminution (antennae) *C. kolensis* DNA were used as reference in calculations of ∆C’ _T_ (Table 1). Since the genome’s size decreases by a factor of 15.6 as a result of CD, the same cell number before and after CD contains different amounts of DNA. Taking this fact into consideration, the factual ratio between the rDNA copy number will differ from the value obtained by 2 ^–∆C’T^ by a factor of 15.6. The same is valid for *C. insignis* ; with its diploid genome being 2.2 times larger than the post-diminution genome of *C. kolensis* (Table 1).

**Determination of the absolute rRNA gene copy number using the calibration curve**

We estimated rRNA gene copy number using a calibration curve. The calibration curve was constructed based on a series of five-fold dilutions of pDNA with pGEM-20b1/ *Pst* I; each copy contained a 183 bp fragment of the 28S rRNA gene. The initial amounts of rDNA templates were determined in relation to 500, 400, 300, and 200 pg of pDNA in the same manner as was done for the calculation using the previous method.

Taking into account the differences in the genome size of the two species, the copy number of rDNA in the diploid genome was determined for each sample of DNA (200 – 500 pg) using data generated from real-time PCR reactions (Table 2). Formula (2) was used for the calculation:


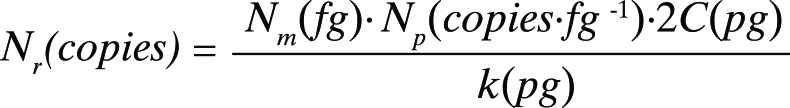
,(2)

where *N _r_* is the 28S rDNA copy number, 2 *С* is the size of the diploid genome, *N _m_* is the experimentally determined initial amount of 28S rRNA gene tamplates, *N _p_* is the number of pDNA molecules per 1 fg of pDNA (see formula 1), and *k * is the amount of the analyzed DNA templates in qPCR.

Thus, the average value of the ratio between the numbers of rRNA genes of prediminuted to post-diminuted genome of *C. kolensis * is 329.94 ± 19.09 (Table 2), while that for the *C. insignis * genomeis 11.73 ± 1.16.

##  DISCUSSION 

The methods for calculating the gene copy number used in this study require that a series of assumptions be made. The main assumption for the 2 ^–∆C’T ^ method is that the reaction is 100% efficient, which is practically unattainable. When using the calibration curve that was constructed using pDNA, it is assumed that during the PCR, pDNA is amplified with the same efficiency as the specimens of *C. kolensis* and *C. insignis* under study. Hence, the observed differences in the ratios between the rDNA copy numbers result directly from the features of the methods used in the calculation.

In this study, it was ascertained that a considerable fraction of rRNA genes were eliminated from the genome of presomatic cells of *C. kolensis * during CD. Along with the almost 16-fold decrease in the genome size, the rRNA gene copy number decreases, as well. Let us note that this is the first description of gene elimination by CD in cyclops. Moreover, no evidence of rDNA elimination in multicellular organisms resulting from CD had previously been described.

**Table 1:** Relative quantification of rRNA genes copy number amounts in prediminuted genomes of *C. kolensis* and soma of *C. insignis* using the 2 ^–∆C’T^ method

Parameters	DNA samples	DNA quantity taken in reaction (pg)				Mean	Standard deviation	Coefficient of variation
500	400	300	200
∆C’_T_	* C. kolensis *before CD	-5.08	-4.99	-4.93	-4.88	-4.97	±0.09	1.7%
	*C. insignis*	-2.96	-2.72	-2.67	-2.63	-2.75	±0.15	5.4%
2^–∆C’T^	* C. kolensis *before CD	33.82	31.78	30.48	29.45	31.38	±1.88	6.0%
	*C. insignis*	7.78	6.59	6.36	6.19	6.73	±0.72	10.7%
Relative number of copies (post diminuted*C. kolensis*serves as a reference sample)	* C. kolensis *before CD	528.01	496.16	475.86	459.78	489.95	±29.41	6.0%
	*C. insignis*	121.48	102.86	99.36	96.64	105.09	±11.22	10.7%

**Table 2:** Absolute quantification of rRNA genes copy number in * C. kolensis * and *C. insignis * using calibration curve

Parameters	DNA samples	DNA quantity taken in reaction (pg)			
500	400	300	200
Mean of template DNA starting quantity (fg) and standard deviation	*C. kolensis *before CD	135±23.2	102±21.3	71.7±18.1	50.3±1.89
	*C. kolensis *after CD	5.99±0.953	4.73±0.553	3.45±0.579	2.56±0.447
	*C. insignis*	37±8.11	25.3±6.13	17.8±3.36	12.7±1.2
rDNA copy number per diploid genome, Nr	*C. kolensis *before CD	1539.86	1454.31	1363.06	1434.35
	*C. kolensis *after CD	4.38	4.32	4.20	4.68
	*C. insignis*	58.62	50.10	47.00	50.30
rDNA copy number ratio –N_r_*(C. kolensis*-before)/N_r_(*C. kolensis*-after)		351.86	336.67	324.46	306.76
rDNA copy number ratio – N_r_(*C. insignis*)/N_r_(*C. kolensis*-after)		13.39	11.60	11.19	10.76

It is possible that the elimination of rRNA genes is attributable to the necessity of aligning the value of the rRNA gene copy number to genome size. A positive correlation between the genome’s size and the rRNA gene copy number was also observed by Prokopovich *et al* . [8]. In a recent elegant experiment with yeast, it was demonstrated that a substantial number of rRNA gene repetitions is important for the general maintenance of the genome’s stability [38]. In particular, it has been ascertained that excessive rDNA copies facilitate sister chromatid association, which is of importance for efficient recombinational repair. Let us note that the elimination of rDNA copies resulting from CD is not proportional to a genome size’s decrease. This is not surprising, since rRNA genes are generally located in the nucleolar organizers of certain chromosomes and are likely to accumulate in one or several clusters [3], instead of distributing uniformly over the genome. Therefore, an accurate mechanism should exist which would make it possible to infallibly cleave the specific fragment of rRNA genes; their loss will not result in functional deficiency of these genes in somatic line cells. Moreover, not all the copies of rRNA genes have to be active; the number of active copies can vary during the ontogenesis. Three states of rDNAs are distinguished. In one state, the active transcription of rRNA genes takes place; in the two other states, genes are not transcribed. However, rRNA genes are prepared at the beginning of the transcription process and, just as in the previous case, have a euchromatin structure. The densely packed nontranscribable rDNA is also sliced out. It has a heterochromatic structure [6]. It is possible that one of these fractions (most probably the heterochromatin fraction) is eliminated from the genome during CD. The possibility of uniform elimination of rDNA copies from all three rDNA fractions should not be excluded, either.

It would be logical to assume that it is functional rDNA copies that predominately remain intact during CD. It has been known that a considerable portion of the rDNA copies of *Drosophila* and other organisms [39, 40] is affected by specific mobile elements (R1, R2); their incorporation results in the inactivation of rDNA copies. It is possible that it is precisely these copies that are eliminated in *C. kolensis* during CD.

Transcription of ribosomal genes is the key element in the regulation of the general level of protein synthesis in a cell [41, 42]. As has been shown in our study, the *C. insignis* genome which lacks CD is more similar to the post-diminuted *C. kolensis * genome, rather than to the pre-diminuted genome, in terms of the rDNA copy number.

 The active rRNA gene expression and synthesis of a substantial number of ribosomes is occurs during early developmental stages. However, CD takes place at the stage of the fourth cleavage division, when the active expression of any genes is yet to begin. At that stage the developmental process occurs at the expense of the storage compounds. Therefore, the excessive genes in pre-diminution blastomeres should not affect the ribosome number in embryonic cells. It is possible that the eliminated copies participate in gamete maturation, where a greater number of ribosomes may be required. 

There that has been an assumption that CD in *C. kolensis * isa developmental stage, during which a transition is made from the cytoplasmic regulation of gene expression during the first cleavage divisions (which is conditioned by the determinants that are already present in the cytoplasm of an unfertilized egg) to the nuclear regulation [1, 29]. Therefore, an initially high abundance of rRNA genes at the pre-diminuted developmental stage of a *C. kolensis * embryo is not possible, whereas the number of rRNA genes in the developing oocyte is over several hundred times larger than the gene number in the post-diminuted genome of somatic cells, and is capable of perfectly determining the higher level of rRNA gene expression in oogenesis, when rRNAs (necessary for the first cleavage divisions) are accumulated.

In conclusion, we would like to note that studying the intra-genomic variation of the rDNA amount in *C. kolensis * as a result of CD is directly linked to understanding the mechanisms of regulation and maintenance of the rDNA copy number in the eukaryotic genome.
